# Ultrasensitive, Selectivity Detection of Mercury Ion Using a Novel Localized Surface Plasmon Resonance Biosensor

**DOI:** 10.3390/s26102967

**Published:** 2026-05-08

**Authors:** Wenyu Xu, Yuanfu Zhang, Yaqi Liu, Lekai Li, Xianfeng Shao, Xinzhi Li, Xueru Chen, Xianxi Zhang

**Affiliations:** Shandong Provincial Key Laboratory of Chemical Energy Storage and Novel Cell Technology, School of Chemistry and Chemical Engineering, Liaocheng University, Liaocheng 252000, China; wenyuhsu@163.com (W.X.); 13181071920@163.com (Y.L.); 18369631897@163.com (L.L.); 18560272659@163.com (X.S.); 15762146499@163.com (X.L.); 18615078097@163.com (X.C.); zhangxianxi@lcu.edu.cn (X.Z.)

**Keywords:** 4-mercaptopyridine-functionalized AuNPs, LSPR sensor, detection of mercury ion

## Abstract

Mercury ion, a highly toxic and bioaccumulative heavy metal pollutant, poses significant risks to human health and ecosystems even at trace concentrations. Therefore, the development of highly sensitive and selective analytical methods for mercury ions is critically important to safeguard environmental integrity and human health. In this work, 4-mercaptopyridine-functionalized gold nanoparticles (4-MPY-AuNPs) were synthesized and subsequently immobilized onto quartz slides to fabricate a localized surface plasmon resonance (LSPR) sensor. Exploiting the selective coordination interaction between the pyridyl nitrogen atoms of 4-MPY and Hg^2+^, this LSPR sensor enables highly specific detection of Hg^2+^. Moreover, injecting a trace amount of 4-mercaptopyridine-functionalized AuNPs into the flow cell triggers the in situ formation of a surface-confined AuNP–Hg^2+^–AuNP sandwich architecture, thereby enhancing the sensor’s sensitivity. Under the optimized conditions, the proposed method exhibited a linear dynamic range of 1 × 10^−9^–6 × 10^−7^ mol L^−1^, with a correlation coefficient (R^2^) of 0.9917 and a limit of detection (LOD) of 3.2 × 10^−10^ mol L^−1^; the LOD of this method is one order of magnitude lower than the LODs reported in contemporary Hg^2+^ detection methods. This method exhibits high selectivity, sensitivity, cost-effectiveness, and is label-free, thereby demonstrating significant potential for environmental applications.

## 1. Introduction

Mercury, a highly toxic heavy metal, poses significant threats to ecosystems and human health even at trace concentrations. Its high mobility and propensity for bioaccumulation enable its entry into the food chain, leading to irreversible adverse effects including neurological impairments, renal dysfunction, and DNA damage [1,2,3,4]. In light of these risks, regulatory bodies such as the WHO and the U.S. EPA have established stringent limits for mercury in drinking water, with a maximum permissible level of 2 ppb (approximately 10 nM) [5,6]. Therefore, the development of analytical methods with exceptional sensitivity and high selectivity for mercury ions is of critical importance for preserving environmental quality and protecting human health Conventional methods for mercury determination, such as atomic absorption spectrometry (AAS) [7,8] and inductively coupled plasma mass spectrometry (ICP-MS) [9,10], are well established for their high sensitivity and reliability. Specifically, cold vapor generation AAS (CV-AAS) [11] offers exceptional selectivity for mercury analysis. ICP-based approaches allow for multi-element analysis with ultra-low detection limits. However, these techniques are often limited by the need for large and expensive instrumentation, laborious sample preparation protocols, and specialized operational expertise, rendering them less practical for routine monitoring or field-based applications. To address these limitations, researchers have developed a suite of innovative strategies, including electrochemical [12,13,14], fluorescence [15], and colorimetric [16,17] approaches, among others, for the detection of mercury ions. Han et al. [18] developed a 4-mercaptopyridine-functionalized electrochemical sensor that enables dual-signal readout using differential pulse voltammetry (DPV) and electrochemical impedance spectroscopy (EIS), attaining a detection limit of 0.358 ng L^−1^. Liu et al. [19] introduced a ratiometric fluorescence probe employing dual-emissive carbon dots (b-CDs and r-CDs) for the highly selective sensing of mercury ions, with a reported detection limit of 5.3 nmol L^−1^. Although fluorescence-based methods offer exceptional sensitivity, the synthesis and purification of the requisite probe molecules are frequently complex and laborious, which may impede their scalability and practical implementation. Colorimetric methods enable naked-eye visualization of detection results, making them well-suited for rapid on-site screening applications. Nevertheless, the colorimetric approach exhibits comparatively low sensitivity, is constrained by the homogeneous solution reaction system, and is susceptible to interference from various matrix factors, including coexisting ions, turbidity, chroma, and pH fluctuations, leading to inadequate selectivity.

In recent years, gold nanoparticles (AuNPs) have emerged as a paradigmatic nanoplatform in modern biosensor design, owing to their finely tunable localized surface plasmon resonance (LSPR) response, exceptional biocompatibility, and highly controllable surface functionalization capabilities [20,21,22,23,24]. For example, Kataria et al. [25] developed a colorimetric Hg^2+^ assay using AuNPs functionalized with 3,5-dimethyl-1-thiocarboxamidepyrazole. Yin et al. [26] developed a colorimetric biosensor for Hg(II) detection based on poly(adenine)-templated DNA-functionalized gold nanoparticles. In this system, specific T-Hg^2+^-T coordination triggers the aggregation of AuNPs, resulting in a distinct color shift from red to blue and achieving a detection limit of 3.75 nM. In short, these AuNP-based colorimetric methods rely on the localized surface plasmon resonance of dispersed AuNPs, which exhibit a characteristic absorption band near 520 nm owing to coherent oscillations of conduction electrons. Upon aggregation, interparticle plasmon coupling induces a redshift of this absorption peak to longer wavelengths. These methods offer distinct advantages, including simple operation, rapid response, and naked-eye readout, making them attractive for on-site screening. However, this approach suffers from several inherent limitations [27,28,29]: (1) detection sensitivity is typically confined to the micromolar range; (2) the aggregation process is highly susceptible to environmental variables—including ionic strength, pH, and coexisting ions—thereby undermining both reproducibility and selectivity; (3) quantitative accuracy is inherently constrained by reliance on endpoint absorbance measurements, which fail to capture kinetic information and are prone to interference from turbidity or nonspecific absorption.

LSPR sensors operate on the principle of enhanced local electromagnetic fields near metal nanoparticle surfaces upon optical excitation, which exhibit high sensitivity to alterations in the surrounding environment [30,31,32]. These methods can detect an immediate increase in thickness of a biomolecular layer on the surface of a substrate caused by a reaction between the target solution component and the receptor layer immobilized on the surface [33]. This sensor utilizes a flow-through configuration, which facilitates the real-time observation of specific binding events between target analytes and recognition molecules immobilized on gold nanoparticle surfaces. Simultaneously, nonspecific ions are efficiently eluted during mobile-phase flushing. In contrast to homogeneous analytical methods, this heterogeneous sensing approach significantly enhances detection selectivity via spatial confinement effects and solid-phase interface screening. Consequently, LSPR sensors are extensively utilized in applications including real-time monitoring of biochemical processes [34,35], clinical diagnostics [36,37,38], and environmental surveillance [39]. For example, Chen et al. [40] described an LSPR sensor designed for the ultrasensitive detection of fibrin, which employed a Y-shaped DNA-homing peptide-doped probe. Nevertheless, the detection of small-molecule analytes, particularly metal ions, remains a significant analytical challenge. This difficulty primarily stems from the inherently weak refractive index changes induced by the binding of low-molecular-weight targets, which often fall below the detection limit of conventional LSPR systems.

Hence, we initially synthesized 4-mercaptopyridine-functionalized AuNPs (4-MPY–AuNPs) and constructed a LSPR sensor utilizing these modified nanoparticles as specific recognition elements for Hg(II). This sensor operates in a flow-through configuration and enables label-free, selective, real-time detection by monitoring transient binding events between target metal ions and the functionalized AuNP surface. It is worth noting that injecting a trace amount of 4-MPY–AuNPs into the flow cell induced the in situ formation of a surface-confined AuNP–Hg(II)–AuNP sandwich architecture, which significantly enhanced the sensitivity of Hg(II) detection. The sensor exhibited robust analytical performance when challenged with real-world samples, such as untreated wastewater, whole blood, and soil extracts, collected directly from environmental, clinical, and industrial settings.

## 2. Materials and Methods

### 2.1. Materials and Apparatus

Chloroauric acid and mercuric chloride were purchased from Sinopharm Chemical Reagent Co., Ltd. (Shanghai, China). Trisodium citrate was obtained from Tianjin No.3 Chemical Reagent Factory (Tianjin, China). 3-Aminopropyltrimethoxysilane (APTMS) and Polyethyleneimine (PEI) were purchased from Shanghai Aladdin Biochemical Technology Co., Ltd. (Shanghai, China). 4-mercaptopyridine (4-MPY) was obtained from TCI Chemical Industry Development Co., Ltd. (Shanghai, China). Quartz glass slide was supplied by Shengyi Quartz Products Co., Ltd (Lianyungang, China).

All chemicals and reagents are of analytical grade. Millipore Milli-Q water (18.2 MΩ cm) was used in all experiments.

The LSPR measurements were performed on an Open SPR instrument from Nicoya Lifesciences Inc. (Toronto, ON, Canada). A Hitachi UH4150 UV-VIS-NIR spectrophotometer (Kyoto, Japan) was employed to acquire the UV-visible absorption spectra. The morphology of the prepared AuNPs was observed using a transmission electron microscope (JEM-2100, JEOL, Tokyo, Japan) and SmartLab 9 kW X-ray powder diffractometer (Rigaku, Tokyo, Japan). Infrared spectra (IR) were recorded on a Shimadzu AIM-9000 instrument (Kyoto, Japan).

### 2.2. Preparation of 4-MPY–AuNPs

All glassware was pre-treated by immersion in aqua regia for 12 h. For AuNPs synthesis, 50 mL of 0.04% aqueous chloroauric acid solution was brought to a vigorous boil, followed immediately by addition of 5.6 mL of a 1% trisodium citrate solution. After the solution turned wine-red, the mixture was kept under reflux with continuous stirring for 15 min. Thereafter, 1 mL of a 1 μmol L^−1^ 4-MPY solution was introduced, and stirring was maintained until the suspension cooled naturally to ambient temperature. The colloidal product was then isolated via centrifugation at 12,074× *g* for 10 min, washed with H_2_O and finally resuspended in H_2_O. The purified AuNPs were stored in light-protected amber vials at 4 °C for future use.

### 2.3. Detection of Hg(II) Based on 4-MPY–AuNPs Colorimetric Method

A total of 300 µL of Hg(II) solution (PB 10 mmol L^−1^, pH = 7.0) at varying concentrations (ranging from 0.01 µmol L^−1^ to 10 mmol L^−1^) was added into 500 µL of the prepared 4-MPY–AuNPs solution (20 nmol L^−1^), and the absorption spectra were subsequently measured.

### 2.4. Preparation of LSPR Sensing Chip

The quartz slides (25 × 19 × 1 mm) were first immersed in aqua regia for 12 h, then thoroughly cleaned and dried. They were subsequently treated with a 10% APTMS methanol solution for 4 h, rinsed with methanol, and soaked in a 1% polyethyleneimine solution for 2 h. After immersion in methanol, the slides were ultrasonically cleaned for 10 min and dried at 60 °C for 2 h. Once cooled to room temperature, 50 μL of the prepared AuNPs solution was applied to the amino-modified quartz slides and allowed to incubate for 2 h at room temperature. The slides were then rinsed with H_2_O. The resulting LSPR chips were stored in PB buffer (10 mmol L^−1^, pH = 7.4) at 4 °C.

### 2.5. Detection of Hg(II) Using LSPR Sensing Chip

The AuNPs chip was first rinsed with deionized water at a flow rate of 20 μL/min until a stable baseline was attained (the flow cell volume is 2.4 mm^3^). Following this, 200 μL of a 0.85 nmol L^−1^ solution of 4-MPY–AuNPs was introduced onto the surface of the chip at the same flow rate. Immediately afterward, 200 μL of mercury ion solution (PB 10 mmol L^−1^, pH = 7.0) was injected at varying concentrations, also at 20 μL/min. Regeneration of the chip was performed using citrate buffer (10 mmol L^−1^, pH 4.0) for 10 min. The corresponding LSPR signal was monitored and recorded using an Open SPR™ instrument (Nicoya Lifesciences, Kitchener, ON, Canada).

### 2.6. Measurement of Equilibrium Dissociation Constant (K_D_)

The AuNPs chip was rinsed with deionized water at a flow rate of 20 μL/min until a stable baseline was achieved. Subsequently, 200 μL of the mercury ion solution was injected at a flow rate of 20 μL/min, and the LSPR signal was recorded. The LSPR data were processed with TraceDrawer software (version 1.9) to perform kinetic analysis and determine the dissociation constant (K_D_).

### 2.7. Selectivity Analysis

The AuNPs chip was first rinsed with deionized water at a flow rate of 20 μL/min until a stable baseline was attained. Following this, 200 μL of a 0.85 nmol L^−1^ solution of 4-MPY–AuNPs was introduced onto the surface of the chip at the same flow rate. Immediately afterward, 200 μL of individual metal ion solutions (10 mmol·L^−1^ PB, pH = 7.0) with a concentration of 100 nmol·L^−1^ were injected, also at 20 μL/min. The tested metal ions included Na^+^, K^+^, Ag^+^, Cu^2+^, Fe^2+^, Co^2+^, Cd^2+^, Ca^2+^, Ba^2+^, Mg^2+^, Al^3+^, Fe^3+^, Cr^3+^, Au(III), Pd(II) and Pt(IV). After each detection, the flow cell was thoroughly cleaned to eliminate residual interference prior to the next measurement.

### 2.8. Real Sample Analysis

A 2 L aliquot of water samples (from the laboratory tap and Dong Lake at Liaocheng University) was first filtered through a 0.45 μm membrane filter, and boiled for 20 min. Subsequently, following the previously described procedure for Hg(II) detection using the LSPR sensing chip, 200 μL of the treated sample was injected onto the chip at a flow rate of 20 μL/min. The corresponding LSPR signal was recorded using an Open SPR™ instrument.

## 3. Results

### 3.1. Preparation and Characterization of 4-MPY–AuNPs

**Figure 1:**
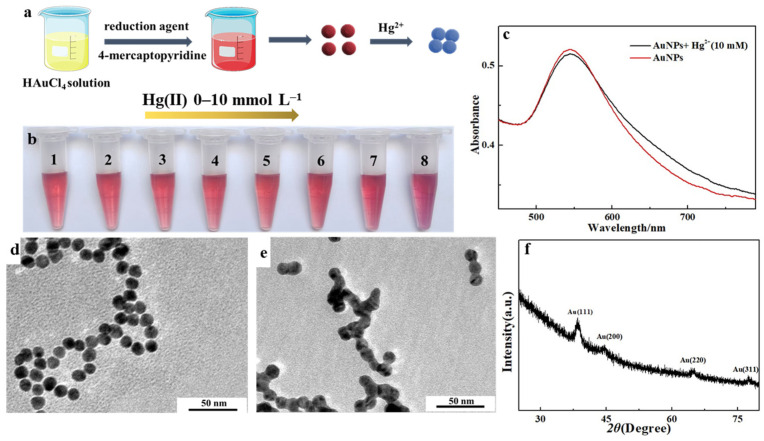
(**a**) Schematic illustration of 4-MPY–AuNPs-based sensing for Hg(II) detection. (**b**) Photograph of 4-MPY-functionalized AuNPs solution upon addition of varying concentrations of Hg(II). (**c**) UV-visible absorption spectra of 4-MPY–AuNPs in the presence of increasing concentrations of Hg(II). (**d**) TEM image of 4-MPY–AuNPs and (**e**) TEM image of 4-MPY–AuNPs in the presence of Hg(II). (**f**) XRD pattern of 4-MPY-functionalized AuNPs.

Figure 1a depicts the synthesis of 4-MPY–AuNPs via a redox reaction, wherein 4-MPY is anchored to the AuNP surface through an Au–S bond. The as-synthesized AuNPs solution exhibits a characteristic wine-red color, with a strong and sharp surface plasmon resonance (SPR) peak centered at 520 nm (Figure S1). Furthermore, the spherical morphology and uniform size of the AuNPs were verified by transmission electron microscopy (TEM) (Figure 1d), showing an average particle diameter of 13 nm and a narrow distribution. The X-ray diffraction (XRD) pattern (Figure 1f) displayed four distinct and sharp peaks at 2θ values of 38.1°, 44.4°, 64.6°, and 77.7°. These peaks correspond to the (111), (200), (220), and (311) crystallographic planes, respectively, characteristic of a face-centered cubic (fcc) gold structure. The correspondence of all identified peaks with the standard powder diffraction patterns confirms the successful synthesis of the material.

### 3.2. Detection of Hg(II) by Utilizing the Hg(II)-Induced Cross-Linking Aggregation of 4-MPY-AuNPs for Colorimetric Sensing

It has been reported that the pyridyl nitrogen atom of 4-MPY selectively coordinates with Hg(II) ions [41]. Therefore, while 4-MPY was modified on the surface of AuNPs, this specific coordination triggers interparticle cross-linking of AuNPs, leading to controlled aggregation of the AuNPs. Leveraging this mechanism, we developed a colorimetric assay based on AuNPs to experimentally validate the feasibility of the proposed Hg(II) recognition strategy. The as-prepared AuNPs dispersion exhibited a characteristic wine-red color due to the LSPR peak at 520 nm. Upon incremental addition of Hg(II), a slight redshift of the LSPR band was observed (Figure 1c), accompanied by a visible color transition from wine-red to purple at 1 mmol L^−1^ Hg(II) (Figure 1b). This redshift reflects a decrease in interparticle distance and the onset of plasmonic coupling, consistent with TEM imaging (Figure 1e), which clearly shows extensive, irreversible agglomeration of AuNPs following Hg(II) addition. Furthermore, in order to illuminate the specificity of identification of 4-MPY–AuNPs, eight metal ions were used to test the response of the assay. While the concentration of metal ions was 1 mmol L^−1^, only Hg(II) induced the characteristic purple coloration (Figure S2), confirming the exceptional selectivity of the AuNPs system toward Hg(II).

### 3.3. Preparation of the 4-MPY–AuNPs-Based LSPR Sensing Chip and Its Application for Hg(II) Detection

**Figure 2:**
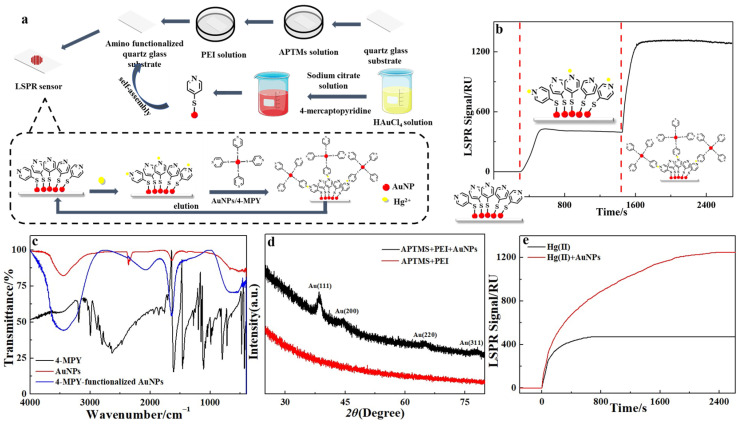
(**a**) Schematic diagram of mercury ion detection. (**b**) LSPR signal profiles for Hg(II) detection. (**c**) Infrared spectra of AuNPs, 4-MPY, and 4-MPY–AuNPs. (**d**) XRD pattern of 4-MPY–AuNPs with cross-linking agent. (**e**) Comparison of LSPR signals in the absence and presence of AuNPs. Experimental conditions: Hg(II) 100 nmol L^−1^, 4-MPY–AuNPs 0.85 nmol L^−1^.

Figure 2a illustrates the detection of Hg(II) with a LSPR sensing chip based on 4-MPY–AuNPs. Firstly, following the aforementioned procedure, 4-MPY–AuNPs were synthesized. Subsequently, 4-MPY-AuNPs were covalently immobilized onto the quartz substrate surface via APTMS-mediated anchoring and further stabilized by PEI-enabled crosslinking, yielding a functional LSPR sensing chip.

4-MPY possesses excellent metal-coordinating capability due to its thiol (–SH) and pyridine nitrogen moieties, which in principle enable coordination with various soft acid metal ions, including Ag^+^, Au(III), Pd(II), and Pt(IV), via the thiol group. In the present sensing platform, however, the thiol group of 4-MPY is fully occupied through immobilization onto the surface of AuNPs, forming stable Au–S bonds. Only the nitrogen atom of the pyridine ring remains available for subsequent selective recognition. Consequently, the actual recognition site at the sensing interface is not the bidentate coordination group of free 4-MPY, but a spatially constrained single site with a defined chemical environment—specifically, the pyridine nitrogen atom. This structure significantly enhances specificity for Hg(II), as the site strongly prefers forming stable coordination with Hg(II) due to its high polarizability and strong affinity for nitrogen, while showing much lower binding affinity for common interfering ions like Cu^2+^, Cd^2+^, and Co^2+^.

Upon injection of Hg(II) onto the LSPR sensing chip, surface-bound 4-MPY ligands selectively coordinate with Hg(II), inducing a red shift in the localized surface plasmon resonance (LSPR) absorption peak and thereby generating a measurable change in the LSPR response signal (Figure 2b). However, the initial LSPR response triggered by Hg(II) is comparatively weak, a limitation attributable to the inherent dependence of LSPR sensitivity on the molecular mass of the bound analyte. Owing to the low molecular weight of Hg(II) (200.59 g mol^−1^), its binding induces only a minor change in the local refractive index, yielding a limited spectral shift. To address this sensitivity constraint, a signal amplification approach was employed through the post-binding introduction of a 4-MPY–AuNPs conjugate solution. As shown in Figure 2b, infusion of this conjugate resulted in a marked amplification of the LSPR signal for Hg(II). This enhancement is due to the formation of a ternary surface-confined sandwich architecture of AuNP–Hg(II)–AuNP, which significantly increases both the effective mass and polarizability at the sensing interface, thereby amplifying the LSPR shift.

FTIR spectroscopy (Figure 2c) was utilized to confirm the successful functionalization of AuNPs with 4-MPY. The IR spectrum of free 4-MPY displayed a characteristic S–H stretching peak at 2500–2800 cm^−1^, along with pyridine ring absorption bands in the 1500–1600 cm^−1^ region. Following conjugation to AuNPs, the S–H stretching peak disappeared entirely, offering direct spectroscopic evidence for thiol bond cleavage and the formation of covalent Au–S bonds. Simultaneously, the pyridine ring absorption bands maintained their positions and relative intensities, indicating that the molecular integrity of 4-MPY was preserved upon immobilization and that the ligand remained anchored to the AuNP surface via the sulfur atom. Furthermore, to ascertain whether the cross-linking process induced structural modifications in the AuNPs, X-ray diffraction (XRD) analysis was performed on the AuNP–crosslinker hybrid. The XRD pattern (Figure 2d) exhibited four distinct diffraction peaks at 2θ values of 38.1 º, 44.4 º, 64.6 º, and 77.7 º, which are indexed to the (111), (200), (220), and (311) crystallographic planes of face-centered cubic (fcc) gold, respectively. Notably, comparison with the XRD profile of pristine AuNPs (Figure 1f) revealed no additional diffraction peaks or detectable peak shifts in the cross-linked assemblies. These observations confirm that the crystalline structure and phase composition of the AuNPs remain unaltered by the cross-linking agent, indicating that the assembly process is governed by surface interactions without perturbing the gold crystal lattice.

To further validate the coordination of Hg(II) with 4-MPY–AuNPs, the binding affinity was evaluated. A 1:2 binding model was employed to derive the dissociation equilibrium constants (K_D_) from curve fitting (Figure S3). The resulting K_D1_ and K_D2_ values were determined to be 1.10 × 10^−8^ M and 8.37 × 10^−9^ M, respectively, indicating a strong binding affinity between Hg(II) and the functionalized nanoparticles. However, the magnitude of the LSPR response was found to depend on both the molecular weight of the analyte and the thickness of the absorption layer. The higher molecular weight correlated directly with stronger binding affinity, while increased absorption layer thickness enhanced the LSPR signal intensity. To improve the sensor’s sensitivity, a 4-MPY–AuNPs solution was introduced onto the chip surface, enabling the formation of a well-defined AuNP–Hg(II)–AuNP (chip) sandwich architecture; this structural design constitutes the key innovation of this work. As shown in Figure 2e, the LSPR response signal in the presence of the 4-MPY–AuNPs solution was more than three times greater than that observed in its absence. To assess the function of the 4-MPY–AuNPs solution, we systematically examined the influence of its concentration on the LSPR response signal. As shown in Figure S4, the LSPR signal exhibited a progressive increase with rising concentrations of 4-MPY–AuNPs, reaching a plateau at 0.85 nmol L^−1^. This behavior can be explained by the initial interaction between a limited quantity of AuNPs and mercury, which is subsequently followed by their reaction with the gold surface on the chip to form a sandwich configuration. In contrast, when the concentration of the 4-MPY–AuNPs solution exceeded 0.85 nmol L^−1^, the LSPR response signal diminished. This attenuation is attributed to the oversaturation of the surface binding sites at excessively high AuNP concentrations, which promotes coordination of Hg(II) ions with immobilized AuNPs rather than facilitating the formation of the intended AuNPs–Hg(II)–AuNPs sandwich structure, thereby reducing the amplification effect. Consequently, a 4-MPY–AuNPs solution at 0.85 nmol L^−1^ was introduced into the chip to enhance signal amplification.

### 3.4. Optimization of Experimental Conditions

To ensure optimal analytical performance, critical experimental parameters were systematically optimized, with the process encompassing three distinct phases: the synthesis of 4-MPY–AuNPs, the fabrication of sensor chips, and the detection of Hg(II) using the proposed LSPR chip.

**Figure 3:**
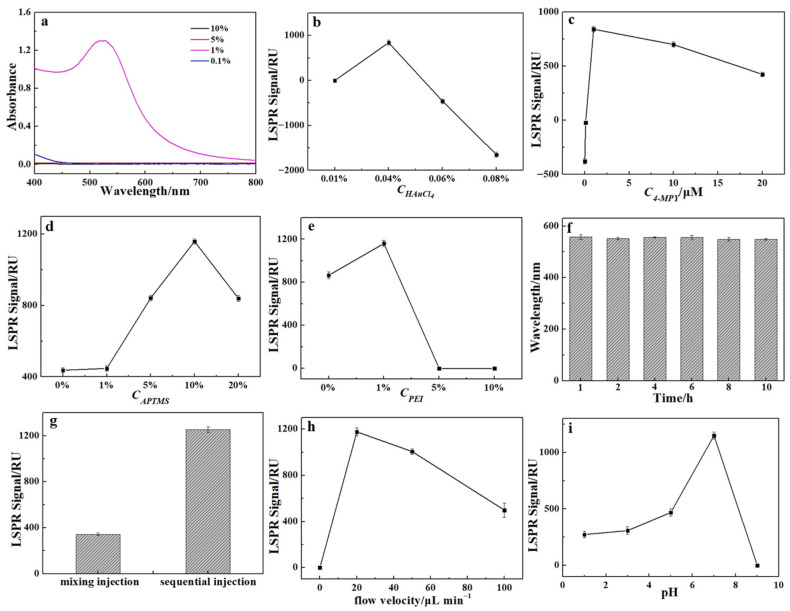
Optimization of key parameters for the LSPR assay. (**a**) UV-visible absorption spectra of 4-MPY-functionalized Au nanoparticles synthesized with varying concentrations of sodium citrate. The influence of (**b**) HAuCl_4_ concentration, (**c**) 4-MPY concentration, (**d**) APTMS concentration, (**e**) PEI concentration, (**f**) APTMS cross-linking duration, (**g**) reagent injection sequence, (**h**) flow rate, and (**i**) pH on the LSPR response signals. Experimental conditions: Hg(II) 100 nmol L^−1^, 4-MPY–AuNPs 0.85 nmol L^−1^.

Firstly, to synthesize 4-MPY–AuNPs exhibiting robust LSPR performance and unambiguous spectral identifiably, the concentrations of sodium citrate, chloroauric acid (HAuCl_4_), and 4-MPY were systematically optimized. The size of the AuNPs was found to depend on the concentrations of sodium citrate and chloroauric acid. As illustrated in Figure 3a, a well-defined absorption peak at 520 nm was observed with 1% (*w*/*v*) sodium citrate, indicating successful AuNP synthesis. Deviations from this optimal concentration—either lower or higher—resulted in the loss of the characteristic plasmonic absorption, leading to the selection of 1% sodium citrate for further experiments. Figure 3b reveals that an insufficient concentration of HAuCl_4_ prevented the formation of AuNPs, whereas a concentration of 0.04% yielded 4-MPY–AuNPs with the strongest signal response for Hg(II) detection. Higher HAuCl4 concentrations produced larger nanoparticles, which compromised the stability of the sandwich assembly and diminished the LSPR signal. Therefore, 0.04% HAuCl_4_ was chosen. As shown in Figure 3c, the optimal LSPR response occurred at 1 µmol L^−1^ 4-MPY. Lower concentrations provided an inadequate number of pyridine rings for Hg(II) coordination, while higher concentrations promoted AuNPs aggregation via nonspecific adsorption, thereby reducing the availability of pyridine rings and attenuating the LSPR signal.

Secondly, 4-MPY–AuNPs were immobilized on quartz slides using APTMS and PEI as crosslinking agents. Consequently, the concentrations of both crosslinkers and the crosslinking duration were systematically optimized (Figure 3d–f and Figure S5). The strongest LSPR response for Hg(II) sensing was obtained at an APTMS concentration of 10%. Lower concentrations led to inadequate assembly of 4-MPY-AuNPs, whereas higher concentrations promoted nanoparticle aggregation and inefficient crosslinking, both attenuating the signal. Thus, 10% APTMS was chosen for subsequent experiments. An optimal PEI concentration of 1% was identified; exceeding this level caused direct aggregation of 4-MPY–AuNPs on the substrate, compromising sensor fabrication. A crosslinking time of 4 h provided the most stable LSPR signal and was selected as the optimal condition.

Thirdly, a direct LSPR-based sensing approach for Hg(II) detection is implemented through the sequential addition of AuNPs and Hg(II) solution onto the functionalized chip surface. The findings reveal that the order in which AuNPs and Hg(II) are injected profoundly affects the analytical sensitivity of the assay. This investigation compared two distinct injection protocols: the sequential delivery of AuNPs and Hg(II) solutions, and the introduction of a pre-mixed solution containing both species. As shown in Figure 3g, the LSPR signal acquired via sequential injection was markedly stronger than that from the pre-mixing approach. This difference arises because pre-mixing facilitates the formation of the sandwich complex in solution prior to injection, leaving no residual Hg(II) available to assemble the sandwich structure on the chip surface and thus resulting in no observable signal amplification. Meanwhile, the injection flow rate significantly affects the formation of the sandwich structure on the chip. Given that the minimum configurable flow rate of the peristaltic pump is 20 μL/min, this value was selected as the initial condition; the flow rate was then incrementally increased at a fixed gradient to systematically evaluate the effect of flow variation on the sensing response performance. It was observed that the LSPR response intensity decreased monotonically with increasing injection flow rate (Figure 3h). This occurred because excessively high flow rates shortened the residence time of Hg(II), thereby impeding complete coordination; concurrently, the elevated shear forces likely destabilized the preformed sandwich structure, resulting in diminished binding affinity and a concomitant signal reduction. Therefore, 20 µL min^−1^ of injection flow rate was selected. Furthermore, the pH of the Hg(II) solution is critical to the detection performance, as it governs both the coordination behavior of Hg(II) and the protonation state of the pyridine groups on the sensor surface. To prevent hydrolysis, a concentrated stock solution of Hg(II) was prepared in 10 mmol L^−1^ HCl. As illustrated in Figure 3i, under acidic conditions, protonation of the pyridine rings diminishes their ability to coordinate Hg(II), yielding a weak signal. At pH 9, hydrolysis of Hg(II) occurs, compromising binding efficiency. Consequently, a phosphate buffer at pH 7 was identified as optimal and was employed for all subsequent experiments.

### 3.5. Analytical Performance of LSPR Sensor for Hg(II) Detection

**Figure 4:**
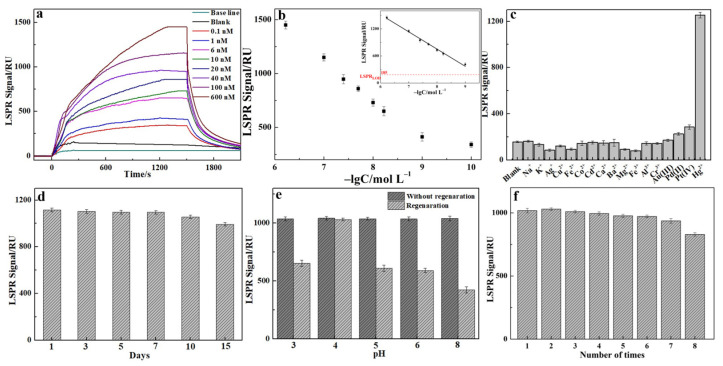
(**a**) LSPR kinetic curves for Hg(II) detection. (**b**) Calibration curve for Hg(II) detection. The inset shows the linear relationship between the LSPR signal and Hg(II) concentration in the range of 1–600 nmol L^−1^. (**c**) The selectivity of the sensor for Hg(II) detection. (**d**) The influence of storage time on LSPR signals. (**e**) The effect of regeneration buffer pH on the LSPR signal of the sensor. (**f**) The effect of regenerated number on LSPR signal of the system. Experimental conditions: Hg(II) 100 nmol L^−1^, citrate buffer 10 mmol L^−1^ pH 4.

Under experimentally optimized conditions, the analytical performance of the LSPR biosensor was systematically assessed using a series of Hg(II) standards covering multiple orders of magnitude in concentration (Figure 4a). The LSPR sensor’s spectral shift displays an exponential dependence on the effective thickness of the Hg(II)-induced absorption layer, in contrast to a linear relationship. To improve data interpretability and ensure uniform representation across the dynamic range, Hg(II) concentrations were subjected to a base-10 logarithmic transformation prior to analysis. As theoretically anticipated, the LSPR response increased monotonically with rising Hg(II) concentration. A highly linear calibration curve (R^2^ = 0.9917) was achieved by plotting the LSPR signal against log_10_[Hg(II)] over the range of 1 to 600 nmol L^−1^, as illustrated in Figure 4b. The regression equation was found to be LSPR Signal = 3775.95 + 378.12 lgC (where C represents the concentration of Hg(II) with the unit of mol L^−1^). The detection limit (LOD) was determined through the following procedure: first, the LSPR-based LOD (LSPR_LOD_) was derived from the expression LSPR_LOD_ = LSPR_Blank_ + 3σ, where LSPR_Blank_ represents the LSPR signal of the blank solution and σ denotes the standard deviation of the blank measurements. Subsequently, using the linear regression equation, lgC_LOD_ was calculated as lgC_LOD_ = (LSPR_LOD_ − 3775.95)/378.12 = −9.50. Finally, the concentration at the detection limit, C_LOD_, was determined to be 0.32 nmol L^−1^ via the relation 10^(lgC_LOD_), confirming the proposed sensor’s capability to reliably quantify trace-level Hg(II) contamination in environmental samples. In contrast to the AuNPs-based colorimetric approach presented herein, the absorbance intensity exhibited a reduction with increasing Hg(II) concentration, as depicted in Figure S6. Nevertheless, the observed change in absorbance was relatively modest, precluding the establishment of a reliable linear correlation. A comparison of this sensor with other State-of-the-Art Hg(II) detection methodologies is presented in Table 1. Relative to existing techniques, the proposed strategy offers distinct advantages including cost efficiency, operational simplicity, and exceptional sensitivity whose LOD is tenfold lower than those reported for most recently published assays.

To evaluate the specificity of the sensor for Hg(II), its response was investigated in the presence of environmentally relevant metal ions which included Na^+^, K^+^, Ag^+^, Cu^2+^, Fe^2+^, Co^2+^, Cd^2+^, Ca^2+^, Ba^2+^, Mg^2+^, Al^3+^,Fe^3+^, Cr^3+^, Au(III), Pd(II) and Pt(IV) tested at a concentration of 100 nmol L^−1^. As illustrated in Figure 4c, the LSPR signal response to Hg(II) was markedly stronger than that to other metal ions, indicating the high selectivity of the sensor for Hg(II). This is attributed to the covalent immobilization of the thiol group of 4-MPY onto the gold substrate, which renders only the pyridine nitrogen atom accessible for selective recognition. Consequently, the sensing interface presents a singular and well-defined recognition site, in contrast to the bidentate coordination geometry characteristic of free 4-MPY. Furthermore, the high polarizability of Hg(II), coupled with their strong affinity for nitrogen atoms, collectively underpins the highly specific binding affinity for Hg(II). Owing to the high polarizability and strong nitrogen affinity of Hg(II), the second-layer 4-MPY-AuNPs can be selectively coordinated by Hg(II), which facilitates the formation of a stable sandwich structure and induces significant LSPR signal amplification. In contrast, other metal ions exhibit weak coordination ability with pyridine nitrogen and cannot bind to the second-layer AuNPs, thus exhibiting negligible LSPR responses.

For the LSPR assay, the sensor chips should be stored in phosphate-buffered saline (PBS, 10 mM, pH 7.4) at 4 °C to preserve the colloidal stability of the gold nanoparticles immobilized on the chip surface. The results indicate that the LSPR signal retained over 95% of its initial intensity after the sensor chip was stored in PBS buffer (10 mM, pH 7.4) for 10 days (Figure 4d), with signal degradation commencing thereafter, demonstrating that the proposed LSPR chip remains stable for at least one week under storage conditions. The regeneration capability of an LSPR sensor represents a crucial parameter for its practical deployment, as it is a direct indicator of the sensing platform’s stability and cost-efficiency. Given the strong chelating interaction between the citrate and Hg (II), citrate buffer was selected as the eluent for regenerating the new chip. When the pH of the citrate buffer is higher than 5 or lower than 3, the regenerated chip exhibits a reduction in response signal exceeding 30% relative to the initial signal in Figure 4e. The response signal remains at the level of the initial signal only when the citrate buffer has a pH of 4. At lower pH, protonation of pyridine (Py) to PyH^+^ competed with Hg(II) coordination, thereby disrupting the sandwich configuration and potentially causing detachment of 4-MPY–AuNPs, which led to signal attenuation upon subsequent injection. At pH ≥ 5, Hg(II) coordination with pyridine was sufficiently stable to prevent efficient regeneration of the chip surface. Furthermore, The LSPR signal response exhibited no significant change over seven consecutive regeneration cycles on the same chip (Figure 4f). In summary, although the LSPR sensor developed in this study supports fewer elution cycles than commercially available alternatives, its unit production cost is only a few RMB, calculated by averaging the total procurement cost of reagents and consumables over the number of chips produced per batch, which is significantly lower than the market price of commercial sensors, which generally exceeds one thousand Yuan.

### 3.6. Detection in Real Samples

In order to elucidate the practicality and reproducibility of the LSPR sensor, real-world water samples were analyzed using the standard addition method. Samples were collected from both the laboratory tap water supply and the lake located on the campus of Liaocheng University. Water samples were spiked with Hg(II) at each of the three specified concentrations. As presented in Table 2, the recoveries for these samples ranged from 96.6% to 108%, indicating that the LSPR sensor holds considerable promise for the practical detection of Hg(II) in real water samples.

## 4. Conclusions

In summary, we have fabricated an ultrasensitive and highly selective LSPR biosensor for Hg(II) detection, utilizing 4-MPY–AuNPs immobilized on a quartz substrate. While conventional LSPR sensors typically exhibit limited selectivity and sensitivity for metal ions, the controlled infusion of trace amounts of 4-MPY–AuNPs into the flow cell induced the on-site formation of a surface-confined AuNP–Hg(II)–AuNP ternary complex, which substantially enhanced Hg(II) sensing performance. This approach enabled the detection of Hg(II) at concentrations as low as 0.32 nmol L^−1^. Furthermore, the sensor exhibits exceptional reusability, with experimental validation confirming its stable performance over seven consecutive cycles. Consequently, the cost per detection has been lowered to a single-digit figure (in Chinese yuan), substantially improving its economic viability for large-scale implementation. Herein, the developed LSPR biosensor demonstrated high specificity and ultra-sensitivity, proving effective for direct analysis of untreated wastewater obtained from the environment.

## Figures and Tables

**Table 1:** Comparison of the proposed approach with other published assays by different methods for Hg(II) detection.

Method	Material	LOD	Linear Range	Reference
Electrochemical	PdNPs@rGO	0.33 μM	1.0–40 μM	[42]
Fluorometry	Si-QDs	0.028 μM	0.5–5.0 μM	[43]
Fluorometry	CT-IONP	7.38 nM	0.02–10 μM	[44]
Colorimetry	AuNPs/MOF	5.4 nM	10–100 nM	[45]
Colorimetry	AuNP@Ag	10 nM	0.1–700 μM	[46]
Fluorometry	C-QDs	42.4 nM	0–5 μM	[47]

**Table 2:** Determination of Hg(II) in water samples using the proposed method and AAS method.

Sample ^a^	Added (nmol L^−1^)	Proposed Method (nmol L^−1^) ^b^	AAS ^c^ (nmol L^−1^)	Recovery (%)
1	5	4.98 (±0.2)	4.96	96.6
	10	10.63 (±0.5)	10.61	106
	20	21.13 (±0.8)	21.1	106
2	5	5.13 (±0.1)	5.10	103
	10	10.78 (±0.5)	10.76	108
	20	21.27 (±1.0)	21.24	106

^a^ Sample 1 was tap water, sample 2 was Dong lake water. ^b^ Average of three replicates ±SD. ^c^ Atomic absorption spectrometry.

## Data Availability

No datasets were generated or analyzed during the current study.

## Supplementary Materials

The following supporting information can be downloaded at: https://www.mdpi.com/article/10.3390/s26102967/s1, Figure S1: UV–vis absorption spectra of 4-MPY-functionalized AuNPs; Figure S2: Photograph of 4-MPY-functionalized AuNPs solution upon addition of varying mental ions; Figure S3: The kinetic curve of the binding of mercury ions to 4-MPY functionalized AuNPs; Figure S4: Effects of 4-mercaptopyridine-functionalized AuNPs concentration on the LSPR response signals; Figure S5: Effects of PEI cross-linking time on the LSPR response signals; Figure S6: The absorbance curve for Hg(II) detection at concentrations ranging from 0 to 10 mmol L^−1^.
