# Supplementary material for: Ultrasensitive, Selectivity Detection of Mercury Ion Using a Novel Localized Surface Plasmon Resonance Biosensor
Source: Sensors (Basel). 2026 May 8;26(10):2967. doi: 10.3390/s26102967 (PMC13211190; doi:10.3390/s26102967)
Supplement: Supplementary file 1 [file sensors-26-02967-s001.zip › sensors-4278267-supplementary.pdf]

Ultrasensitive, Selectivity Detection of Mercury Ion Using a Novel Localized Surface Plasmon Resonance Biosensor

Wenyu Xu, Yuanfu Zhang *, Yaqi Liu, Lekai Li, Xianfeng Shao, Xinzhi Li, Xueru Chen and Xianxi Zhang

Shandong Provincial Key Laboratory of Chemical Energy Storage and Novel Cell Technology,

School of Chemistry and Chemical Engineering, Liaocheng University, Liaocheng 252000, China; wenyuhsu@163.com (W.X.); 13181071920@163.com (Y.L.); 18369631897@163.com (L.L.);

18560272659@163.com (X.S.); 15762146499@163.com (X.L.); 18615078097@163.com (X.C.);

zhangxianxi@lcu.edu.cn (X.Z.)

* Correspondence: zhangyuanfu@lcu.edu.cn

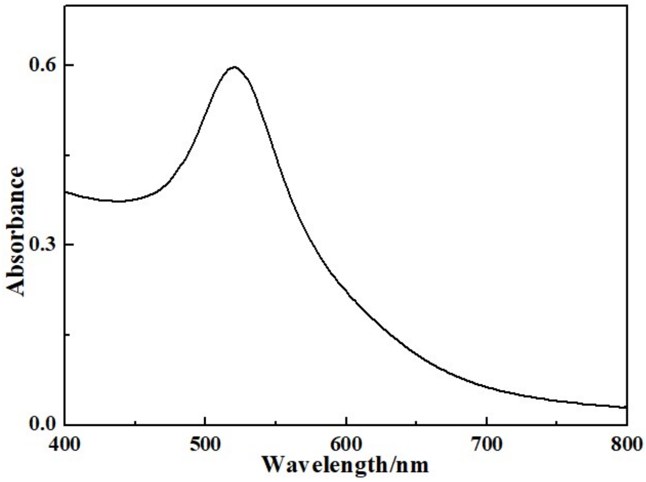

Figure S1 UV–vis absorption spectra of 4-MPY-functionalized AuNPs.

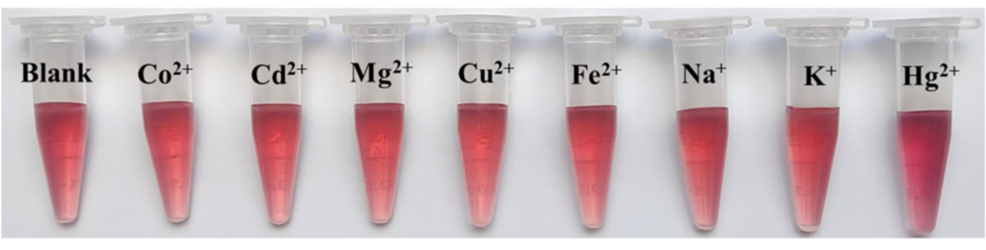

Figure S2 Photograph of 4-MPY-functionalized AuNPs solution upon addition of varying mental ions.

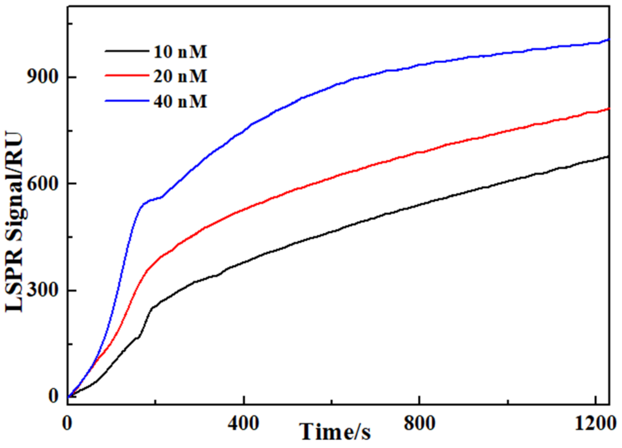

Figure S3 The kinetic curve of the binding of mercury ions to 4-MPY functionalized AuNPs.

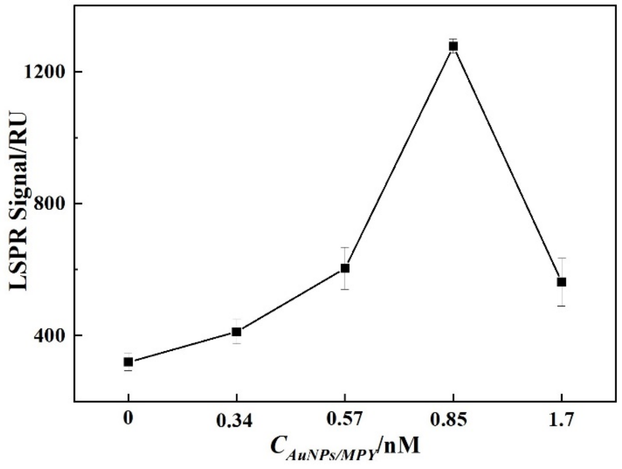

Figure S4 Effects of 4-mercaptopyridine-functionalized AuNPs concentration on the LSPR response signals.

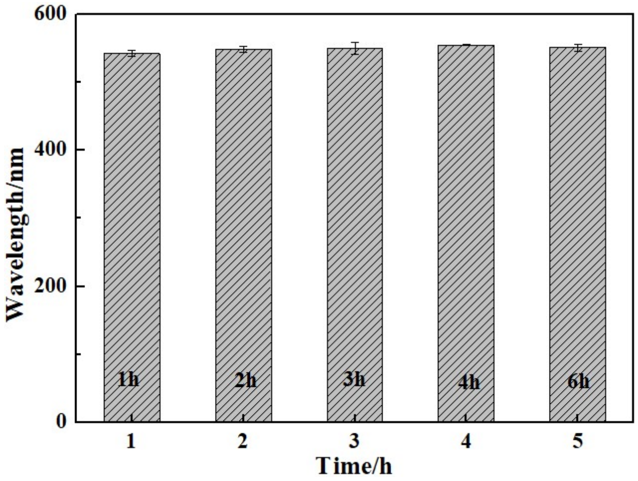

Figure S5 Effects of PEI cross-linking time on the LSPR response signals.

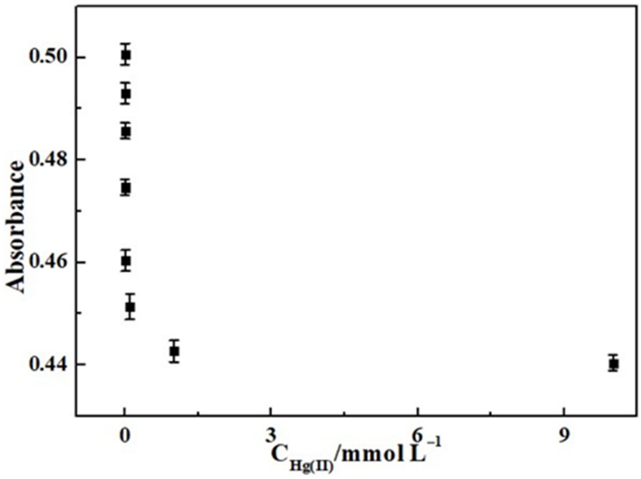

Fig. S6 The absorbance curve for Hg(II) detection at concentrations ranging from 0 to 10 mmol L^{-1}.
